# Neutrophil Extracellular Traps (NETs) Promote Pro-Metastatic Phenotype in Human Breast Cancer Cells through Epithelial–Mesenchymal Transition

**DOI:** 10.3390/cancers12061542

**Published:** 2020-06-11

**Authors:** Karina Martins-Cardoso, Vitor H. Almeida, Kayo M. Bagri, Maria Isabel Doria Rossi, Claudia S. Mermelstein, Sandra König, Robson Q. Monteiro

**Affiliations:** 1 Institute of Medical Biochemistry Leopoldo de Meis, Federal University of Rio de Janeiro, Rio de Janeiro 21941 590, Brazil; kmcardoso@bioqmed.ufrj.br (K.M.-C.); vhluna@bioqmed.ufrj.br (V.H.A.); 2 Institute of Biomedical Sciences, Federal University of Rio de Janeiro, Rio de Janeiro 21941 590, Brazil; kayobiomed14@histo.ufrj.br (K.M.B.); idrossi@hucff.ufrj.br (M.I.D.R.); mermelstein@ufrj.br (C.S.M.); sandra@icb.ufrj.br (S.K.); 3 Clementino Fraga Filho University Hospital, Federal University of Rio de Janeiro, Rio de Janeiro 21941 913, Brazil

**Keywords:** epithelial–mesenchymal transition, neutrophil extracellular traps, breast cancer, metastasis

## Abstract

Neutrophil extracellular traps (NETs) have been associated with several steps of tumor progression, including primary growth and metastasis. One of the key features for the acquisition of the metastatic ability is the epithelial–mesenchymal transition (EMT), a complex cellular program. In this study, we evaluated the ability of isolated NETs in modulating the pro-metastatic phenotype of human breast cancer cells. Tumor cells were treated with isolated NETs and then samples were generated for cell migration, quantitative RT-PCR, western blotting, immunofluorescence, and flow cytometry assays. RNA-seq data from The Cancer Genome Atlas (TCGA) database were assessed. NETs changed the typical epithelial morphology of MCF7 cells into a mesenchymal phenotype, a process that was accompanied by enhanced migratory properties. Additional EMT traits were observed: increased expression of N-cadherin and fibronectin, while the E-cadherin expression was repressed. Notably, NETs positively regulated the gene expression of several factors linked to the pro-inflammatory and pro-metastatic properties. Analyses of TCGA data showed that samples from breast cancer patients exhibit a significant correlation between pro-tumoral and neutrophil signature gene expression, including several EMT and pro-metastatic factors. Therefore, NETs drive pro-metastatic phenotype in human breast cancer cells through the activation of the EMT program.

## 1. Introduction

Breast cancer is the most prevalent with the highest mortality rate in women worldwide [1]. Immunohistochemical markers, as well as genomic data, allow classifying breast cancer into subtypes that are biologically distinct and behave differently concerning therapeutic response and clinical outcome [2,3]. Breast cancer subtypes include luminal A, luminal B, human epidermal growth factor receptor-2 positive/estrogen receptor-negative (HER2+/ER−), and triple-negative (which includes basal-like). These subtypes are associated with distinct patterns of metastatic spread with significant differences in survival after relapse, in which luminal A and triple-negative represent the less and the more aggressive subtypes, respectively [4,5].

The ability of cancer cells to disseminate from primary tumors to form new tumor colonies in distant tissues is defined as metastasis, one of the hallmarks of cancer [6]. Acquisition of the metastatic capacity is a complex process that may involve an intricate cellular program named epithelial–mesenchymal transition (EMT). EMT is driven by a set of transcriptional factors, including Snail (also known as SNAI1), Slug (also known as SNAI2), Twist-related protein 1 (TWIST1), zinc-finger E-box-binding homeobox 1 (ZEB1) and ZEB2, that regulate gene expression alterations which culminate with enhanced tumor cell migration, invasion, and metastatic properties [7,8]. EMT program is strongly influenced by stromal cells in the tumor microenvironment, which include endothelial cells, fibroblasts, inflammatory, immune cells, and others [9,10,11].

Among the immune cells, neutrophils are the most abundant and the first inflammatory cells recruited to the sites of tissue damage and infection [11,12]. Several lines of evidence indicate that tumor-associated neutrophils are important players in cancer progression [12,13]. More recently, it has been proposed that neutrophils may influence the tumor properties through the release of neutrophil extracellular traps (NETs) [14,15,16,17]. Primarily described as an antimicrobial mechanism, NETs are composed of a double-stranded DNA decorated with neutrophil nuclear and granular proteins, such as citrullinated histones, myeloperoxidase, metalloproteinases, and elastase [18]. Subsequent studies have demonstrated that NETs have several pro-tumoral capabilities, including the ability to sequester circulating tumor cells and contribute to metastasis [19,20], to support primary tumor growth [21,22], to modulate the pro-inflammatory tumor microenvironment [23,24] and to establish the cancer-associated prothrombotic state [25,26,27]. It is unclear, however, whether NETs may influence EMT to support tumor progression.

In the present study, we evaluated the ability of isolated NETs in modulating the pro-metastatic phenotype of human breast cancer cells. Incubation of isolated NETs with the luminal cell line, MCF7, altered the epithelial morphology into a mesenchymal phenotype. In accordance with the acquisition of the mesenchymal phenotype, MCF7-treated cells showed enhanced migratory properties. Morphological changes were accompanied by enhanced gene expression of the EMT-related transcriptional factors, *ZEB1* and Snail (*SNAI1*). Notably, the treatment of MCF7 cells with NETs increased the expression of N-cadherin and fibronectin, while the E-cadherin expression was repressed. NETs positively regulated gene expression of several factors linked to the pro-inflammatory and pro-metastatic properties of breast cancer cells, including interleukin-1β (IL-1β/*IL1B*), interleukin-6 (IL-6/*IL6*), interleukin-8 (IL-8/*CXCL8*), *CXCR1*, matrix metalloprotease-2 (MMP-2/*MMP2*), *MMP9*, and *CD44*. Further analyses of data from The Cancer Genome Atlas (TCGA) showed that samples from breast cancer patients exhibit a significant correlation between neutrophil signature and pro-tumoral genes, including several EMT and pro-metastatic factors. Our results suggest that NETs released in the primary tumor may contribute to the acquisition of metastatic properties during breast cancer progression. Taken together, the modulation of NETs formation during tumor progression might represent an attractive therapeutic target to decrease the metastatic spread.

## 2. Results

### 2.1. NETs Alter the Morphology and Enhance the Migratory Pattern in MCF7 Cells 

**Figure 1:**
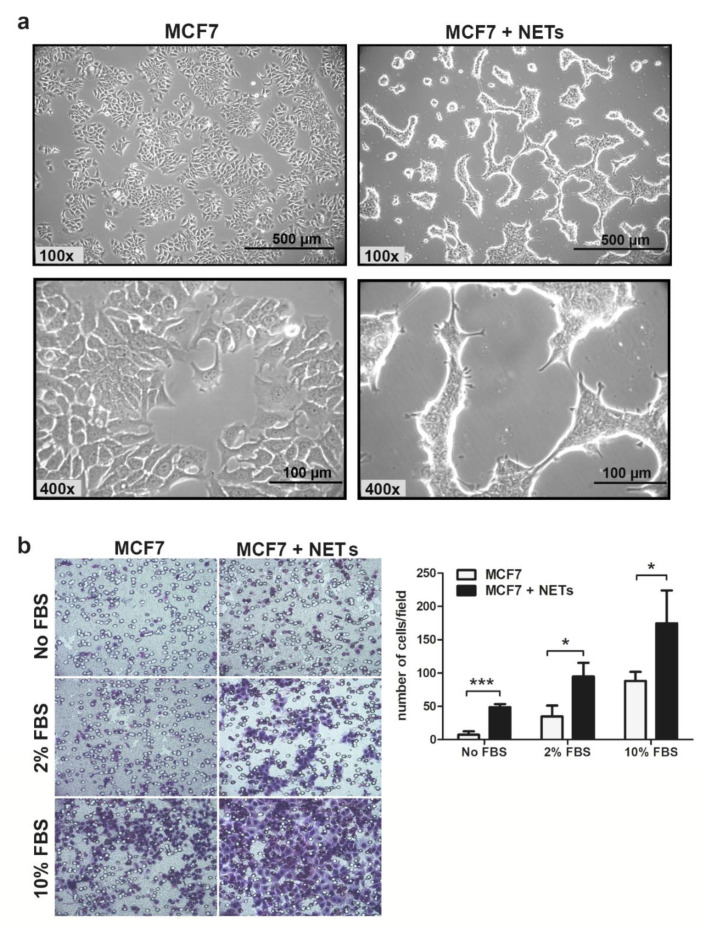
Neutrophil extracellular traps (NETs) alter cell morphology and enhance MCF7 migration in vitro. (**a**) Representative images of MCF7 cells that were cultured for 16 h in the absence (left) or the presence (right) of NETs (500 ng/mL). Magnification 100× and 400×, scale bar 500 µm and 100 µm, respectively. (**b**) Tumor cell migration was evaluated employing the Boyden chamber assay. MCF7 cells that were cultured for 16 h in the absence or the presence of NETs (500 ng/mL) were seeded in the upper chamber (5 × 10^4^ cells/well) and further allowed to migrate for 20 h. As chemoattractant, medium supplemented with fetal bovine serum (FBS) (2% or 10%) was used in lower chambers. Representative images of the migration assay are shown on the left panel (200× magnification). Migrated cells were quantified, and results are shown on the right panel. Data are presented as mean ± SD from three independent experiments. Statistical analysis of each condition was evaluated by unpaired *t*-test. Significance was assumed for * *p* < 0.05, *** *p* < 0.001.

The MCF7 cell line, which has been classified as a luminal subtype [28], displays an epithelial phenotype, with polyhedral form, and can form islets in vitro. Incubation of MCF7 cells with NETs for 16 h promoted drastic morphological changes (Figure 1a).

After 8 h of treatment with NETs, MCF7 cells began to acquire a more elongated fibroblast-like shape, presenting an expressive amount of membrane protrusions. We also noticed the loss of cell adhesion to the cell culture flasks after treatment with NETs. Previous findings showed that NETs increase the migratory pattern of tumor cells including colorectal, lung carcinoma, and lymphoma [15,19,29]. Then, we sought to evaluate the effect of NETs on the migratory behavior of MCF7 cells. Treatment of MCF7 cells with NETs enhanced the tumor cell migration either in the absence or in the presence of 2 or 10% fetal bovine serum (FBS) used as a chemoattractant (Figure 1b). Thus, isolated NETs promoted MCF7 migration in all conditions tested in this study.

### 2.2. NETs Promote EMT in Breast Cancer Cells

**Figure 2:**
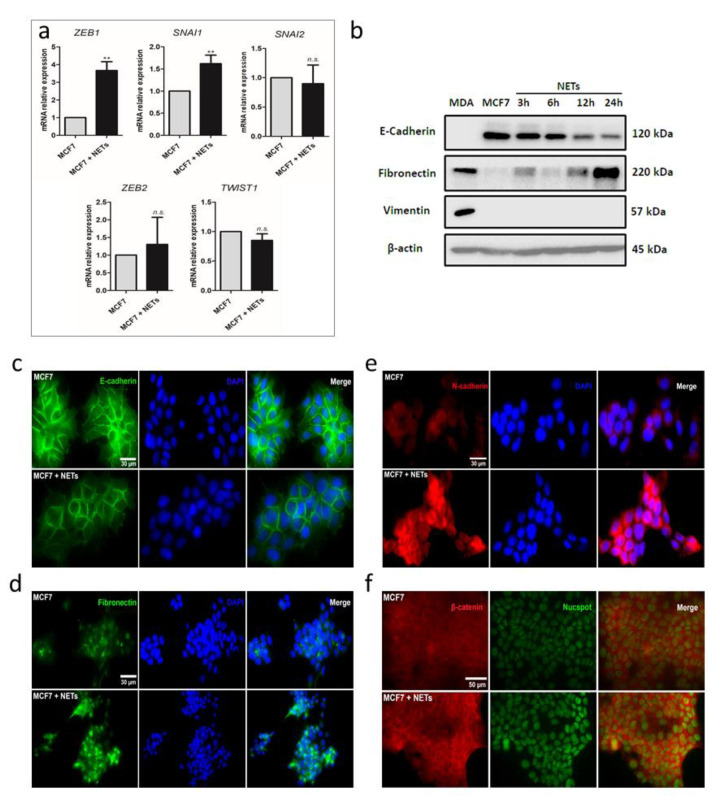
NETs promote epithelial–mesenchymal transition (EMT) in MCF7 cells. (**a**) Gene expression of EMT transcription factors was analyzed by quantitative RT-PCR. *GAPDH* was used as the reference gene. Relative expression of mRNA was calculated using the ΔΔCT method. Columns represent means ± SD of a minimum of three independent experiments. Unpaired *t*-test was applied for statistical analysis. ** *p* < 0.01 and *n.s.*, no significance. (**b**) Western blot analysis of the EMT markers protein levels (E-cadherin, fibronectin, and vimentin) in MCF7 cells (1 × 10^6^) treated with NETs (500 ng/mL) for 3 to 24 h. β-actin was used as a loading control and MDA-MB-231 (MDA) was used as a mesenchymal cell model. Representative image from two independent experiments. Immunocytochemistry analysis of (**c**) E-cadherin (green, magnification 630×, scale bars 30 μm); (**d**) fibronectin (green, magnification 400×, scale bars 30 μm); (**e**) N-cadherin (red, magnification 630×, scale bars 30 μm); and (**f**) β-catenin (red, magnification 630×, scale bars 50 μm) in MCF7 cells that were cultured for 16 h in the absence (above) or the presence (below) of NETs (500 ng/mL). Nuclei were stained with 4’,6-Diamidino-2-Phenylindole (DAPI) (blue) or NucSpot (green) and merged images are shown on the right panels.

Changes in cell morphology induced by NETs seemed a typical EMT process [30]. To investigate the possibility of a transition from epithelial to mesenchymal features induced by NETs in MCF7 cells, we next evaluated the expression of transcriptional factors known to regulate the EMT process. Quantitative RT-PCR analyses showed a significant increase in the expression of ZEB1 (*ZEB1)* and Snail (*SNAI1*) genes upon treatment of MCF7 cells with NETs (Figure 2a). No changes in the expression pattern of *ZEB2*, Slug (*SNAI2*), or Twist1 (*TWIST1)* were observed. The EMT process is marked by the loss of epithelial markers, such as E-cadherin, along with the enhancement of expression of mesenchymal markers, such as N-cadherin, fibronectin, and vimentin [7]. We further employed western blotting to evaluate the protein levels of E-cadherin, fibronectin, and vimentin. As expected, luminal-like MCF7 cells express E-cadherin and failed to express fibronectin and vimentin while basal-like MDA-MB-231 cells present a mesenchymal profile with no E-cadherin and high fibronectin and vimentin expression patterns (Figure 2b and Figure S1a–d). Interestingly, E-cadherin levels gradually reduced over time in MCF7 cells upon treatment with NETs (Figure 2b and Figure S1a,d). A similar result was observed upon treatment of the HER2+ breast cancer cell line, HCC 1954 (Figures S1e and S2), both at the protein and gene expression levels. On the other hand, fibronectin levels were progressively increased in MCF7 cells over the incubation time (Figure 2b and Figure S1b,d). Vimentin expression, which is usually not observed in MCF7 cells, appeared to be not modulated upon incubation with NETs (Figure 2b and Figure S1c,d).

Changes in the expression pattern of E-cadherin and fibronectin were confirmed by immunofluorescence assays (Figure 2c,d and Figure S3). The typical E-cadherin downregulation observed in the EMT process is usually accompanied by an increase in the N-cadherin expression, a process known as “cadherin switching”. Here, we also employed immunofluorescence to demonstrate an increase in the N-cadherin expression pattern in MCF7 cells cultured in the presence of NETs (Figure 2e and Figure S3).

E-cadherin is a cell surface protein that may associate with the multifunctional protein, β-catenin, at the cell membrane. β-catenin commonly acts as a signal transducer of the canonical Wnt pathway, also related to EMT [31,32]. Earlier works suggest that E-cadherin can physically sequester β-catenin at the cell membrane. Thus, E-cadherin-associated β-catenin represents a reserve pool of β-catenin that can potentially feed into Wnt signaling activity [33]. In this context, immunofluorescence assays revealed higher expression of β-catenin in MCF7 cells treated with NETs, as compared to untreated cells (Figure 2f and Figure S3). Together, these results support the capacity of NETs to promote EMT in the breast cancer cell line, MCF7.

### 2.3. Stem Cell Markers are Modulated by NETs

**Figure 3:**
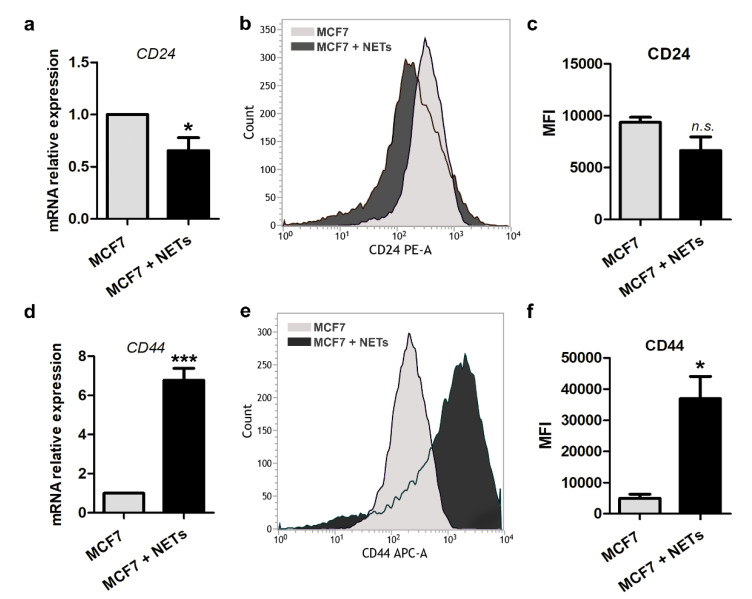
Cancer stem cell markers are regulated by NETs. Gene expression of *CD24* (**a**) and *CD44* (**d**) was analyzed by quantitative RT-PCR in MCF7 cells that were cultured for 16 h in the absence (gray bar) or the presence (black bar) of NETs (500 ng/mL). *GAPDH* was used as the reference gene. The relative expression level of the mRNA was calculated using the ΔΔCT method. Values represent means ± SD of three independent experiments. Representative histograms of flow cytometry analysis of CD24 (**b**) and CD44 (**e**). Graphic representation of relative mean of fluorescence intensities (MFI) of phycoerythrin (PE)-labeled CD24 antibody (**c**) and allophycocyanin (APC)-labeled CD44 antibody (**f**). Data shown are from two independent experiments. Unpaired *t*-test was applied for statistical analysis. Significance was assumed for * *p* < 0.05, *** *p* < 0.001; *n.s.*, no significance.

There is strong evidence that the EMT program is associated with the maintenance of cancer stem cells (CSCs) in solid tumors [34]. In breast cancer, the combined expression of CD44 and CD24, commonly reveals enrichment of the CD44^−^CD24^+^ and CD44^+^CD24^Lo/−^ cell phenotypes in luminal and basal-like breast cancer cell lines, respectively [35,36]. We then investigated if isolated NETs could interfere with stem cell features of MCF7 cells by analyzing the expression of the cell surface markers, CD44 and CD24. As seen in Figure 3, NETs promoted a significant reduction in *CD24* gene expression as well as a trend to decreased cell surface protein expression in MCF7 cells, according to flow-cytometric analysis (Figure 3a–c).

On the other hand, MCF7 cells treated with NETs showed significant enrichment in the CD44 marker, as evaluated by quantitative RT-PCR and flow cytometry (Figure 3d–f). Changes in the expression pattern of CD24 and CD44 markers in NETs-treated MCF7 cells lead us to suggest that along with EMT activation, NETs may promote enrichment in cells with CSC-like features.

### 2.4. NETs Induce a Pro-Inflammatory Response in Breast Cancer Cells

**Figure 4:**
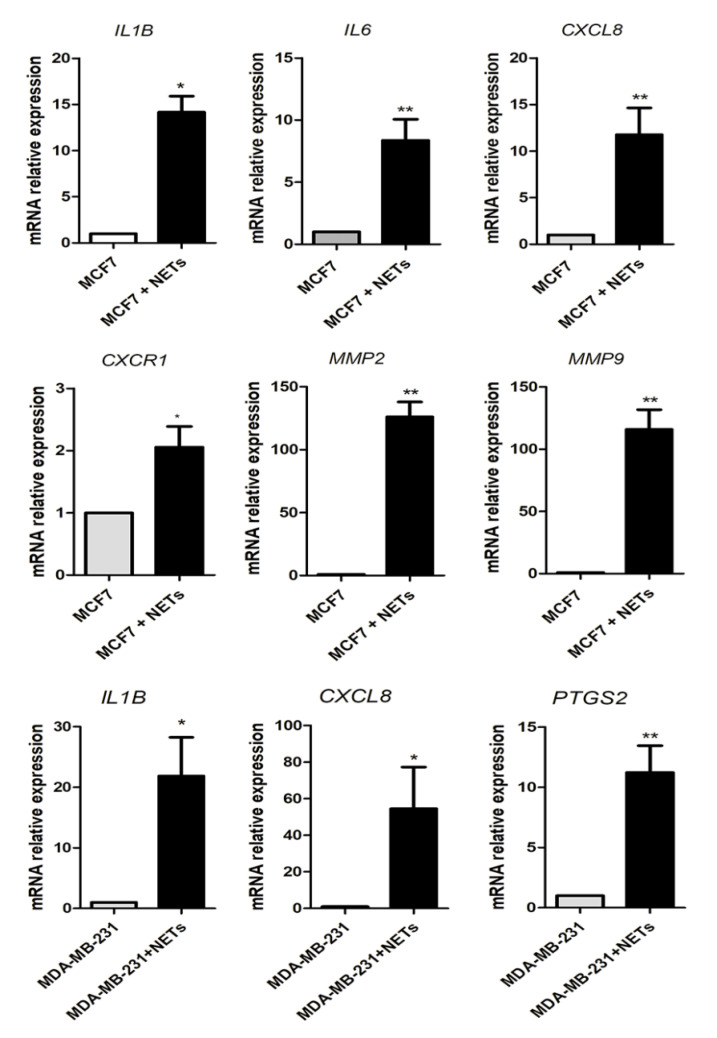
Pro-tumoral and pro-inflammatory mediators are regulated by NETs. MCF7 cells (5 × 10^5^) were starved and further cultured for 16 h in the absence (grey bar) or the presence (black bar) of NETs (500 ng/mL). Genes analyzed: interleukin-1β (IL-1β/*IL1B*), interleukin-6 (IL-6/*IL6*), interleukin-8 (IL-8/*CXCL8*), *CXCR1*, matrix metalloprotease-2 (MMP-2/*MMP2*), and *MMP9*. MDA-MB-231 cells (5 × 10^5^) were cultured for 3 h in the absence or the presence of NETs (500 ng/mL). Genes analyzed: *IL1B*, *CXCL8*, and cyclooxygenase-2 (COX-2/*PTGS2*). Gene expression was evaluated by quantitative RT-PCR using the ΔΔCT method. *GAPDH* was used as the reference gene. Columns represent means ± SD of three independent experiments. Statistical analysis was performed using unpaired *t*-test. * *p* < 0.05 and ** *p* < 0.01.

The activity of the EMT-related transcriptional factors has been linked with the production of pro-inflammatory cytokines that play key roles in the metastatic process [37]. In this context, we next evaluated the ability of NETs in modulating the expression of a set of cytokines described to be crucial for breast cancer development. As shown in Figure 4, quantitative RT-PCR analysis revealed a significant increase in the gene expression of IL-1β/*IL1B* (~15-fold), *IL6* (~10-fold), and IL-8/*CXCL8* (~10-fold). The upregulation of *CXCL8* expression in MCF7 cells was accompanied by the induction of *CXCR1* expression (~2-fold increase), which encodes for a major IL-8 receptor (Figure 4). We further evaluated the impact of NETs on MMPs gene expression, since these enzymes regulate the remodeling of the extracellular matrix, thus favoring invasion and metastasis [9,38]. Remarkably, the expression of *MMP2* and *MMP9* was ~100-fold higher in NETs-treated MCF7 cells as compared to the untreated cells (Figure 4). As seen with MCF7 cells, incubation of HCC 1954 with NETs enhanced *MMP9* expression (Figure S2b). We also employed isolated NETs to treat the basal-like MDA-MB-231 cell line, known to secrete high levels of IL-1β and IL-8. Treatment of MDA-MB-231 cells upregulated *IL1B* and *CXCL8* gene expression, as well as cyclooxygenase-2 (COX-2/*PTGS2)* (Figure 4). Together, these results indicate that NETs induce a pro-inflammatory response in breast cancer cells.

### 2.5. Neutrophil-Related Genes Correlate with Pro-Tumoral and EMT Genes in Breast Cancer Patients

**Figure 5:**
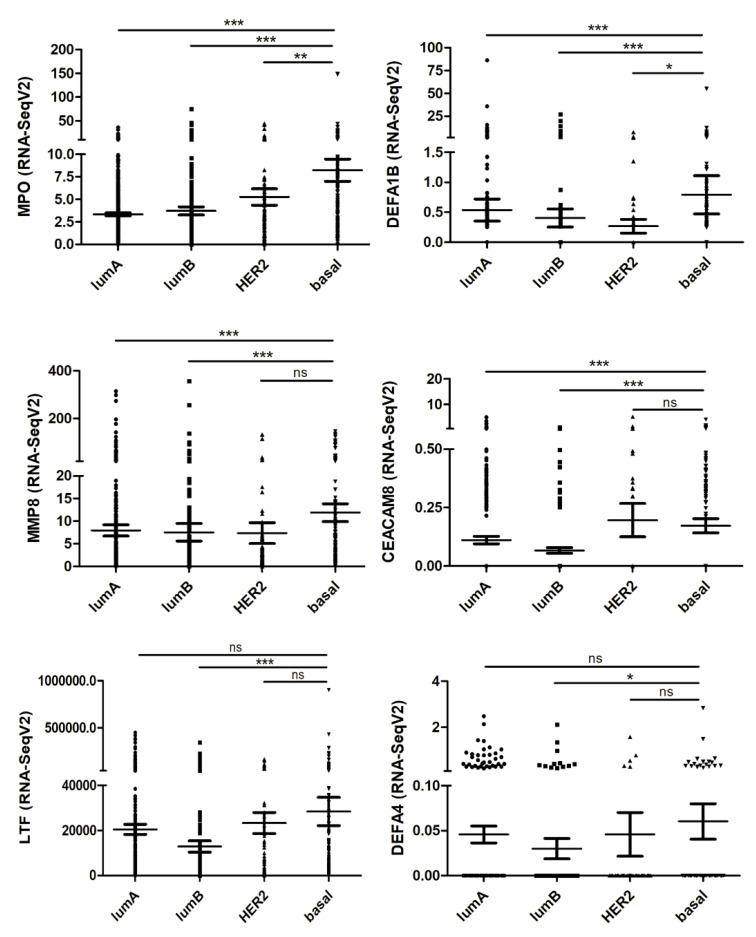
Analysis of neutrophil-related signature genes in the different breast cancer subtypes. RNA seq values (Fragments Per Kilobase Million, FPKM) of 1100 breast cancer samples, deposited in The Cancer Genome Atlas (TCGA) database, were stratified into Luminal A (lumA), Luminal B (lumB), HER2+ (HER2), and Basal subtypes. Genes analyzed: *MPO* (myeloperoxidase), *DEFA1B* (a-defensin 1B), *MMP8* (MMP8), *CEACAM8* (CD66b), *LTF* (lactotransferrin), and *DEFA4* (a-defensin 4). The Mann–Whitney U test was used to test for statistical significance. * *p* < 0.05, ** *p* < 0.01, *** *p* < 0.001, and n.s.: no significance.

To investigate the relevance of our in vitro findings for cancer patients, we used transcriptome data deposited in TCGA database. For this purpose, we first analyzed a set of genes defined as a neutrophil-related signature in the different breast cancer subtypes. These neutrophil-related genes (*MPO*, *DEFA1B*, *MMP8*, *CEACAM8*, *LTF*, and *DEFA4*) were identified and evaluated in a previous study [39]. The *MPO* gene encodes myeloperoxidase, an abundant enzyme in the neutrophil azurophilic granules, which has microbicidal activity through the generation of hypochlorous acid [40]. *DEFA1B* and *DEFA4* encode α-defensins found in azurophil granules of neutrophils. These defensins are small cationic peptides that promote the permeabilization and disruption of cell membranes, killing pathogens [41]. Matrix metalloproteinase-8 (*MMP8*) is an endopeptidase mainly produced by neutrophils. When neutrophils are activated, MMP-8 is released from intracellular granules and cleaves some extracellular matrix proteins, such as collagen, as well as other substrates [42]. CEACAM8, also known as CD66b, is a glycoprotein that plays a role in cell adhesion. CD66b is exclusively expressed on human granulocytes and is recognized as a granulocyte activation marker [43]. *LTF* gene is a member of the transferrin gene family and its protein product, lactotransferrin, is found in the secondary granules of neutrophils. Lactotransferrin released by neutrophils acts as a first-line defense against pathogens through the chelation of iron [44]. As seen in Figure 5, most of the neutrophil-related genes were increasingly expressed from luminal A to basal breast cancer subtypes. This observation agrees with an enhanced neutrophil accumulation in more aggressive breast cancer subtypes [45].

Next, we analyzed the neutrophil-related signature gene expression versus genes encoding pro-inflammatory and pro-metastatic factors (Table 1). We found a positive correlation between neutrophil genes and several pro-tumoral factors that were upregulated in vitro. *CXCL8, CXCR1, IL1B, IL6, MMP2,* and *MMP9* showed a positive correlation with at least 3 out of 6 neutrophil signature genes. We have also analyzed EMT-related genes with the neutrophil signature genes (Table 1). We noticed a positive correlation between the neutrophil signature genes with Snail (*SNAI1)* and β-catenin *(CTNNB1)* genes. As expected, the E-cadherin *(CDH1)* gene expression showed a negative correlation with the neutrophil signature genes, since NETs decreased E-cadherin expression in MCF7 cells. *ZEB1*, fibronectin (*FN1*) and N-cadherin *(CDH2)* correlations were inconclusive. No correlation between the expression of *CD24* and *CD44* genes with the neutrophil signature genes was observed, possibly reflecting the heterogeneity of CSC markers in the primary tumors.

## 3. Discussion

Inflammation is one of the hallmarks of cancer [6]. The presence of leukocytes in the tumor microenvironment is well described and is extremely dynamic during the disease progression. Several lines of evidence support a role for the sustained chronic inflammation in promoting the tumor aggressiveness, including the metastatic potential. Among the immune cells found in the tumor microenvironment, neutrophils have been pointed out as important mediators of tumor progression [12,13,46]. More recently, neutrophil extracellular traps (NETs) have been associated with several steps of tumor progression, including primary growth and metastasis [19,20,21,22].

One of the key mechanisms supported by the immune/inflammatory microenvironment is the epithelial–mesenchymal transition (EMT) [37]. Key features during the EMT process include the loss of epithelial markers, such as E-cadherin, along with the enhancement of expression of mesenchymal markers, such as N-cadherin, fibronectin, and vimentin. This process has been pointed as a dynamic gradient of loss and gain of cellular features and there are several pieces of evidence for the existence of intermediate stages, wherein both mesenchymal and epithelial markers might be co-expressed [7,8] This is in part explained by the concerted action of different transcription factors that modulate the EMT features, in which Snail and ZEB1, which are strong epithelial repressors, seem to be more activated in the intermediate EMT stages [7]. Herein, we observed that NETs promote a significant decrease in the E-cadherin expression by MCF7 cells, with minor changes in the vimentin expression pattern. This was accompanied by increased ZEB1 (*ZEB1)* and Snail *(SNAI1)* gene expression, while no changes in the expression pattern of ZEB2 (*ZEB2*), Slug (*SNAI2*) or Twist1 (*TWIST1)* were observed. In this context, vimentin expression is regulated by Slug in breast cancer models [47]. On the other hand, Huang and co-workers [48] have shown that some ovarian carcinoma cell lines exhibit intermediate EMT states presenting low E-cadherin and high vimentin expression patterns [48]. Interestingly, other EMT inducers, such as epidermal growth factor (EGF) and transforming growth factor-beta (TGF-β), were able to induce vimentin expression in MCF7 cells within 24 h of treatment [49,50]. These data suggest that the mechanism triggered by NETs seems to be slightly different from the other inducers and/or that the NET-evoked EMT occurs in a partial way. The chronic effect of NETs on EMT induction after prolonged treatments deserves further investigation.

Aberrant activation of the Wnt/β-catenin signaling pathway has been associated with several aspects of cancer biology, including tumor initiation, EMT, and metastasis [31]. As a result of the excessive Wnt/β-catenin signaling, β-catenin accumulates in the cytoplasm or within the nucleus of tumor cells, serving as a transcriptional factor, along with other partners, of pro-tumoral genes [31]. Moreover, Kim and colleagues [51] provided data showing that cell membrane-bound β-catenin evokes pro-tumoral responses by enhancing the signaling of growth factor receptors such as the epidermal growth factor receptor (EGFR) [51]. Therefore, β-catenin may exhibit pro-tumoral functions regardless of its subcellular location. Herein it was observed that treatment of MCF7 cells with NETs significantly enhanced β-catenin expression although showing a minor impact on the subcellular location of this protein. Whether NETs-induced changes in the β-catenin expression pattern accounts for the upregulation of pro-tumoral factors have yet to be evaluated.

The EMT program has been correlated with cancer stem cell (CSC) traits, including the expression of stem cell-associated antigens, enhanced chemotherapy resistance, and self-renewal properties [34,52,53]. Among the CSC surface markers, CD44 and CD24 phenotype have been widely employed in breast cancer research [35,36]. CD44 is a cell-surface glycoprotein receptor that recognizes several ligands including extracellular matrix components, such as hyaluronic acid, osteopontin, metalloproteinases, and others [54]. CD44 has been associated with migration and metastasis, being upregulated in the triple-negative breast cancer subtype [54]. Remarkably, CSC subpopulations that exhibit the CD44^+^CD24^Lo/−^ phenotype usually display increased tumorigenic properties and a higher capacity to metastasize [55]. Here, we show that the treatment of MCF7 cells, which typically exhibits the CD44^Lo/−^CD24^+^ phenotype, upregulates the gene and protein expression levels of CD44. It remains to be determined whether these changes parallel with the acquisition of additional CSC features, including additional CSC markers, enhanced tumorigenic properties, and drug resistance.

The major components of NETs (histone, DNA, and granule proteins) are recognized as damage-associated molecular patterns (DAMPs). DAMPS can be recognized through the Toll-like receptors (TLRs). For example, extracellular histones can activate TLR2 and TLR4, while TLR9 is a cell surface receptor of CpG motifs in DNA [56,57]. All TLR signaling pathways culminate in the activation of the transcription factor nuclear factor-kappa B (NF-κB), which controls the expression of several inflammatory cytokine genes [58]. Herein, we observed that the treatment of breast cancer cell lines with NETs upregulates the expression of several pro-inflammatory genes, including IL-8 (*CXCL8*), *IL6*, IL-1β (*IL1B*), and *CXCR1*. Furthermore, bioinformatics tools and chromatin immunoprecipitation assays identified many NF-κB binding sites along with the promoters of SNAI1, SNAI2, ZEB2, and TWIST1 genes [59]. Indeed, NF-κB is essential for EMT and metastasis in a model of breast cancer [60]. In this same line, inflammatory factors in the tumor microenvironment, including TGF-β, IL-6, IL-1β, IL-8, and others can induce EMT [61,62,63]. On the other hand, EMT-transcription factors can modulate inflammation during the EMT process. For example, Katsura and colleagues [64] have shown that knockdown of *ZEB1* in MDA-MB-231 cells decreases the in vitro production of IL-6 and IL-8 [64]. Interestingly, we observed a significant increase in ZEB1 (*ZEB1*) and Snail (*SNAI1*) gene expression by MCF7 cells that were treated with NETs. Together, these data suggest an important linkage between inflammation and EMT signaling in breast cancer cells. Indeed, pro-inflammatory cytokine expression has also been associated with malignant progression and poor prognosis in breast carcinomas [65].

The DNA integrity of NETs has been pointed out as an essential condition for promoting some of their biological activities. Therefore, treatment with DNase, which efficiently degrades NETs, attenuates the development and progression of liver metastases in a murine model of colorectal cancer [15]. Similar antimetastatic effects have been observed in hepatocellular carcinoma and breast cancer models [20,23]. Moreover, the degradation of NETs substantially reduces cancer-associated thrombosis in neutrophilia-related breast and pancreas cancer models [26,66]. Interestingly, we observed that the digestion of NETs with DNase had a minor impact on tumor cell migration as well as in the *CXCL8* and *MMP9* gene expression (Figure S4). Therefore, we believe that, under our experimental conditions, DNA integrity is dispensable for the effect of NETs towards MCF7 cells.

The presence of neutrophils in primary tumors has been correlated with poor prognosis in human cancer [67]. Thus, increased infiltration of intratumoral neutrophils was associated with unfavorable survival and recurrence in several cancer types, including hepatocellular carcinoma, non-small-cell lung cancer, cervical cancer, and others [67]. More recently, it was reported an enhanced neutrophil accumulation in more aggressive breast cancer subtypes [45]. Here, we analyzed transcriptome data from breast cancer patients and showed a positive correlation between neutrophil signature genes and several pro-tumoral factors that were upregulated in vitro upon treatment with NETs, including pro-inflammatory and EMT-related factors. Remarkably, the contribution of neutrophil for the EMT process has been previously suggested in different cancer types, including lung adenocarcinoma and ovarian cancer [68,69].

## 4. Materials and Methods

### 4.1. Cell Culture

Breast cancer cell lines (MCF7, HCC 1954, and MDA-MB-231) were from the Rio de Janeiro Cell Bank (Rio de Janeiro, RJ, Brazil). Cells were maintained in DMEM (Dulbecco’s Modified Eagle Medium, Thermo Fisher Scientific, Waltham, MA, USA) supplemented with 10% fetal bovine serum (FBS) (Cultilab, Campinas, Brazil) and 1% penicillin/streptomycin (Thermo Fisher Scientific) at 37 °C in 5% CO_2_ atmosphere. For all experiments, after seeding, cells were starved for 10 h before treatment with NETs.

### 4.2. Neutrophils Isolation and NETs Obtention

Venous blood from healthy donors was collected in sodium citrate tubes. Neutrophils were purified from whole blood using Histopaque-1077 based (Merck, Darmstadt, Germany) density gradient centrifugation. Isolated neutrophils were stimulated with 500 nM Phorbol 12-myristate 13-acetate (Merck, Darmstadt, Germany) for 4 h. NETs were isolated following a previously described procedure [70], resuspended in sterile phosphate-buffered saline (PBS), and quantified using NanoDrop Lite Spectrophotometer (Thermo Fisher Scientific). Isolated NETs were kept at 4 °C for no more than 24 h. This protocol followed ethical standards and was approved by an institutional committee (Clementino Fraga Filho University Hospital, Federal University of Rio de Janeiro) under registry 82933518.0.0000.525.

### 4.3. Migration Assay

Boyden chamber assay was used to evaluate tumor cell migration employing 8 μm pore polycarbonate membranes (Neuro Probe, Gaithersburg, MD, USA). MCF7 cells (5 × 10^4^) were cultured in the absence or the presence of NETs (500 ng/mL) for 16 h. Cells were further resuspended and seeded to the upper chambers into 50 μL serum-free medium. DMEM medium in the absence or the presence of 2 or 10% FBS was added in the lower compartment. After 20 h of incubation at 37 °C in 5% CO_2_, non-migrated cells on the upper surface of the membrane were removed and the membrane was fixed and stained using Fast Panoptic Staining (Laborclin, Nova Iguacu, Brazil). The average number of migrated cells was calculated from ten random fields counted per condition.

### 4.4. Quantitative RT-PCR

5 × 10^5^ cells were washed twice with PBS and starved in serum-free medium for 10 h followed by treatment with NETs (500 ng/mL). After 16 h, cultured cells were washed twice with PBS to remove NETs, and total RNA was extracted using TRIzol Reagent (Thermo Fisher Scientific, Waltham, MA, USA). From each sample, 1 µg of RNA was submitted to DNase I treatment and reverse transcription PCR. Next, real-time PCR was performed on cDNA with SYBR Green Real-Time PCR Master Mix (Thermo Fisher Scientific, Waltham, MA, USA), using the StepOnePlus Real-Time PCR System (Thermo Fisher Scientific). All reagents and primers were purchased from Thermo Fisher Scientific and showed reaction efficiencies between 90–110%. The primer sequences are shown in the Table S1. Gene expression was normalized using *GAPDH* as the reference gene. To analyze the relative fold change, we employed the 2^−ΔΔCT^ method.

### 4.5. Western Blot

1 × 10^6^ cells were starved in serum-free medium and treated with NETs (500 ng/mL) for 3, 6, 12, or 24 h. After the treatment, cells were washed, lysed and proteins were quantified using the Lowry method (DC protein assay, Bio-Rad, Hercules, CA, USA). Protein lysates (30 μg) were run on 6–10% polyacrylamide gel electrophoresis under denaturing conditions in the presence of sodium dodecyl sulfate and transferred onto PVDF membranes (GE Healthcare, Sao Paulo, Brazil). Membranes were blocked and incubated overnight, at 4 °C, with the following primary antibodies against: E-cadherin (1:10,000; #61082; BD Biosciences, San Jose, CA, USA), fibronectin (1:750; #F3648; Merck, Darmstadt, Germany), vimentin (1:500; #M0725; DakoCytomation, Glostrup, Denmark) or β-actin (1:1000; #8457; Cell Signaling Technology, Danvers, MA, USA). Then, the membranes were incubated with HRP-conjugated secondary antibodies (DakoCytomation) for 1 h, at room temperature, and immunoblots were detected using the ECL reagent (GE Healthcare, Sao Paulo, Brazil).

### 4.6. Immunofluorescence Microscopy

MCF7 cells (2.5 × 10^5^) were seeded on 22 mm-Aclar plastic coverslips (Pro-Plastics Inc., Linden, NJ, USA) previously coated with rat-tail collagen. After treatment with NETs (500 ng/mL) for 16 h, cells were fixed with 4% paraformaldehyde diluted in PBS (pH 7.4), permeabilized with PBS containing 0.5% Triton X-100 and incubated with primary antibodies against: β-catenin (1:50, #C-2206, Sigma Chemical Co, Saint Louis, MO, USA), E-cadherin (1:50, #04-1103, Millipore, Burlington, MA, USA), fibronectin (1:50, #F-6140, Sigma Chemical Co, Saint Louis, MO, USA) or N-cadherin (1:50, #C-3865, Sigma Chemical Co, Saint Louis, MO, USA) for 1 h at 37 °C. Cells were washed and incubated for 1 h at 37 °C with secondary antibodies Alexa Fluor 546 or Alexa Fluor 488 (1:100), all purchased from Thermo Fischer Scientific. Nuclei were labeled with 0.1 μg/mL DAPI (Thermo Fisher Scientific) or NucSpot (1:500, Biotium, Hayward, CA, USA) for 5 min. Slides were mounted in ProLong Gold antifade reagent (Molecular Probes, Eugene, OR, USA) and examined in an Axiovert 100 inverted microscope (Carl Zeiss, Oberkochen, Germany). Images were acquired with an Olympus DP71 digital camera (Olympus, Shinjuku City, Japan). The overall fluorescence intensity was quantified using the ImageJ software (NIH, Bethesda, MD, USA), and results were expressed as a percentage, considering untreated cells as 100%.

### 4.7. Flow Cytometry Analysis

After treatment with NETs (500 ng/mL) for 16 h, cells were harvested and counted. A suspension with 1 × 10^6^ cells/mL in serum-free medium was washed twice with flow cytometry buffer (PBS containing 0.01% sodium azide and 3% FBS). Next, conjugated antibodies were added, and cells were incubated for 30 min on ice. For this assay, mouse anti-human CD24 antibody conjugated with phycoerythrin (Clone ML5; Thermo Fisher Scientific) and rat anti-CD44 conjugated with allophycocyanin (Clone IM7; Thermo Fisher Scientific) were used. Flow cytometry acquisition was performed using a FACSCanto II with FACSDiva software (BD Biosciences, San Jose, CA, USA). The analysis was done using FlowJo software (BD Biosciences, San Jose, CA, USA) and the mean fluorescence intensity (MFI) of CD24 and CD44 was evaluated.

### 4.8. Gene Expression Correlation Analysis

Transcriptome data from 1100 breast cancer samples available at The Cancer Genome Atlas (TCGA, Firehose Legacy Study) were accessed using the cBioPortal [71,72]. The cBioPortal platform provided visualization, analysis, and the ability to download large-scale cancer genomics data sets. In our study, we analyzed the correlation between the expression of a previously defined set of neutrophil-related genes (*DEFA4*, *DEFA1B*, *MMP8*, *CEACAM8*, *LTF*, and *MPO*) [39] and the expression of genes involved in inflammation, metastasis, EMT, and stemness in this database.

### 4.9. Statistical Analysis

For statistical analysis was applied the GraphPad Prism 5 (GraphPad Software, San Diego, CA, USA). Data are shown as mean ± standard deviation. The unpaired *t*-test was used to determine a significant difference between MCF7 cells and MCF7 cells treated with NETs in quantitative RT-PCR, flow cytometry, and migration assay. The details of the statistics are indicated in the figure legends. The correlation of the RNA-seq values (FPKM) was statistically analyzed by the non-parametric Spearman test. Results were considered statistically significant when *p*-value ≤ 0.05.

## 5. Conclusions

Together, the present study shows, for the first time, that isolated NETs promote epithelial–mesenchymal transition (EMT) in cultured breast cancer cells. Our findings suggest that NETs released in the primary tumor may influence the acquisition of metastatic properties during breast cancer progression. Overall, the modulation of NETs formation in the tumor microenvironment might represent an attractive therapeutic target to decrease or even prevent the metastatic spread.

## Figures and Tables

**Table 1:** Correlation analysis between neutrophil signature genes and pro-tumoral genes in human breast cancer samples from The Cancer Genome Atlas (TCGA).

Genes	*CEACAM8*	*DEFA1B*	*DEFA4*	*LTF*	*MMP8*	*MPO*
Interleukin-1β (*IL1B*)	r = 0.0767 *p* = 0.0109	r = 0.116 *p* = 0.0001	r = 0.0655 *p* = 0.0297	r = 0.255 *p* < 0.0001	r = 0.225 *p* < 0.0001	r = 0.183 *p* < 0.0001
Interleukin-6 *(IL6)*	r = 0.0983 *p* = 0.0011	r = 0.197 *p* < 0.0001	r = 0.124 *p* < 0.0001	r = 0.232 *p* < 0.0001	r = 0.208 *p* < 0.0001	r = 0.314 *p* < 0.0001
Interleukin-8 (*CXCL8*)	r = 0.0445 *p* = 0.14	r = 0.141 *p* < 0.0001	r = −0.0277 *p* = 0.359	r = 0.0963 *p* = 0.0014	r = 0.39 *p* < 0.0001	r = 0.156 *p* < 0.0001
CXCR1 *(CXCR1)*	r = 0.0242 *p* = 0.422	r = 0.117 *p* < 0.0001	r = 0.0879 *p* = 0.0035	r = 0.131 *p* < 0.0001	r = 0.0677 *p* = 0.0248	r = −0.008 *p* = 0.788
MMP-2 *(MMP2)*	r = −0.00765 *p* = 0.800	r = 0.0114 *p* = 0.705	r = 0.0351 *p* = 0.245	r = 0.173 *p* < 0.0001	r = 0.286 *p* < 0.0001	r = 0.154 *p* < 0.0001
MMP-9 *(MMP9)*	r = 0.012 *p* = 0.692	r = 0.114 *p* = 0.0002	r = 0.120 *p* < 0.0001	r = 0.0315 *p* = 0.297	r = 0.408 *p* < 0.0001	r = 0.21 *p* < 0.0001
Snail (*SNAI1*)	r = 0.0959 *p* = 0.0014	r = 0.162 *p* < 0.0001	r = 0.0522 *p* = 0.0834	r = 0.179 *p* < 0.0001	r = 0.281 *p* < 0.0001	r = 0.211 *p* < 0.0001
ZEB1 *(ZEB1)*	r = −0.0491 *p* = 0.103	r = −0.0773 *p* = 0.0103	r = 0.0183 *p* = 0.544	r = 0.0921 *p* = 0.0022	r = 0.132 *p* < 0.0001	r = 0.0499 *p* = 0.0982
E-cadherin (*CDH1*)	r = −0.041 *p* = 0.174	r = −0.0728 *p* = 0.0157	r = −0.0796 *p* = 0.0083	r = −0.194 *p* < 0.0001	r = −0.0473 *p* = 0.117	r = −0.144 *p* < 0.0001
Fibronectin (*FN1*)	r = −0.0507 *p* = 0.0927	r = −0.0792 *p* = 0.0086	r = −0.0464 *p* = 0.124	r = −0.0149 *p* = 0.621	r = 0.384 *p* < 0.0001	r = −0.0309 *p* = 0.307
N-cadherin (*CDH2*)	r = 0.0134 *p* = 0.657	r = −0.0217 *p* = 0.472	r = −0.045 *p* = 0.136	r = −0.102 *p* = 0.0007	r = 0.275 *p* < 0.0001	r = 0.0302 *p* = 0.316
β-catenin (*CTNNB1*)	r = −0.0022 *p* = 0.941	r = −0.0476 *p* = 0.115	r = −0.0004 *p* = 0.99	r = 0.196 *p* < 0.0001	r = 0.176 *p* < 0.0001	r = 0.0633 *p* = 0.0359
CD24 *(CD24)*	r = 0.030 *p* = 0.317	r = 0.0319 *p* = 0.291	r = −0.00795 *p* = 0.792	r = −0.0114 *p* = 0.706	r = 0.0796 *p* = 0.0083	r = 0.0605 *p* = 0.0447
CD44 *(CD44)*	r = −0.0254 *p* = 0.399	r = 0.0458 *p* = 0.129	r = 0.0443 *p* = 0.142	r = 0.014 *p* = 0.642	r = 0.0157 *p* = 0.602	r = 0.0578 *p* = 0.0555

Grey: No correlation; green: Positive correlation; red: Negative correlation. r = coefficient of correlation.

## Supplementary Materials

The following are available online at https://www.mdpi.com/2072-6694/12/6/1542/s1, Figure S1: Uncropped blots for analysis of EMT markers, Figure S2: Effects of NETs on HER2+ breast cancer cells, Figure S3: Quantitative analysis of immunocytochemistry assays for EMT markers in NETs-treated MCF7 cells, Figure S4: The pro-tumoral effects of NETs are independent of DNA integrity, Table S1: qRT-PCR primer sequences.
