# Supplementary material for: Neutrophil Extracellular Traps (NETs) Promote Pro-Metastatic Phenotype in Human Breast Cancer Cells through Epithelial–Mesenchymal Transition
Source: Cancers (Basel). 2020 Jun 11;12(6):1542. doi: 10.3390/cancers12061542 (PMC7352979; doi:10.3390/cancers12061542)
Supplement: Supplementary file 1 [file cancers-12-01542-s001.pdf]

cancers

MDPI

Article

Neutrophil Extracellular Traps (NETs) Promote Pro-Metastatic Phenotype in Human Breast Cancer Cells through Epithelial-Mesenchymal Transition

Karina Martins-Cardoso, Vitor H. Almeida, Kayo M. Bagri, Maria Isabel Doria Rossi, Claudia S. Mermelstein, Sandra König and Robson Q. Monteiro

Supplementary Materials

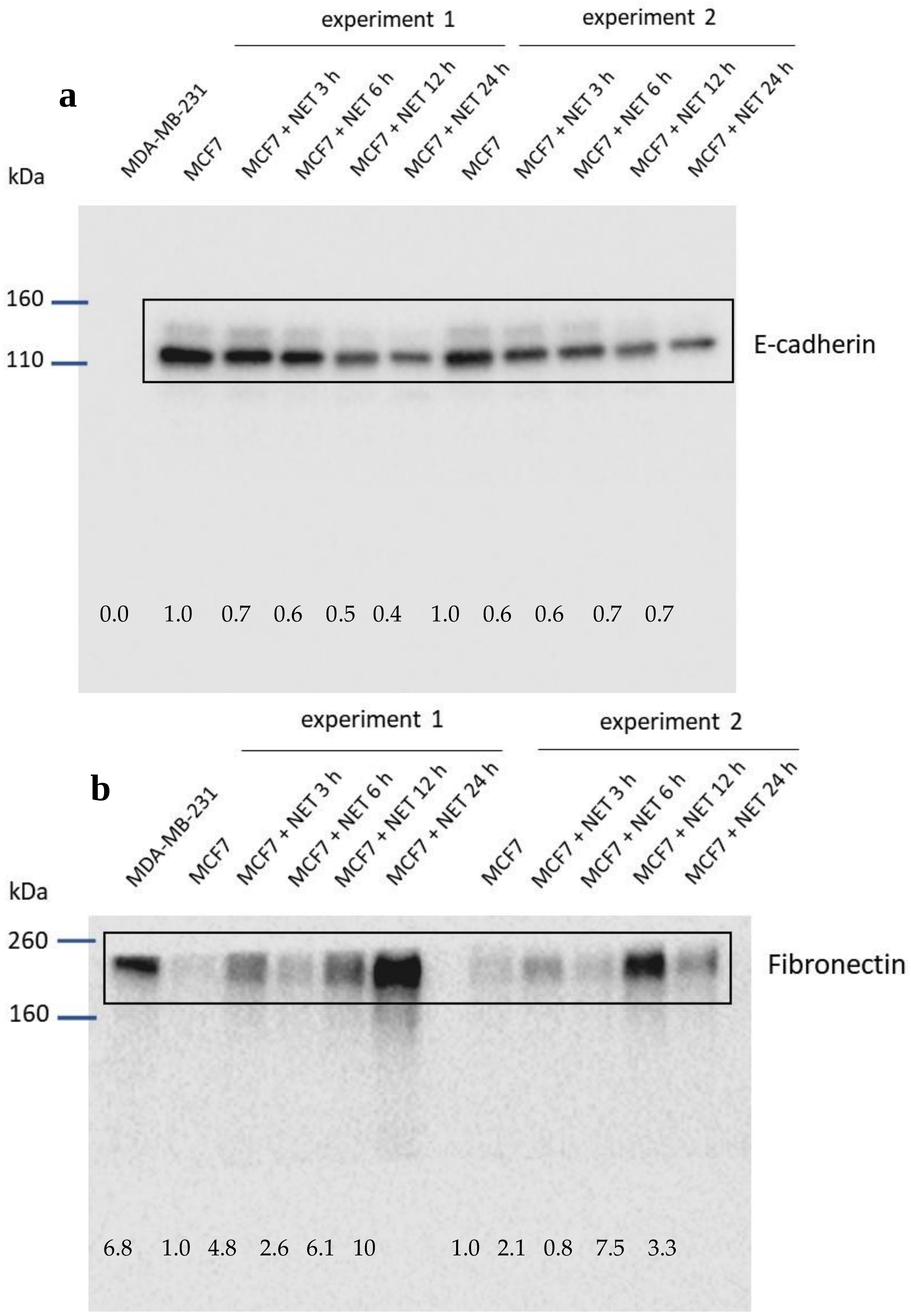
experiment 1
experiment 2
a
MDA-MB-231
MCF7
MCF7 + NET 3 h
MCF7 + NET 6 h
MCF7 + NET 12 h
MCF7 + NET 24 h
MCF7
MCF7 + NET 3 h
MCF7 + NET 6 h
MCF7 + NET 12 h
MCF7 + NET 24 h
kDa
160
110
E-cadherin
0.0 1.0 0.7 0.6 0.5 0.4 1.0 0.6 0.6 0.7 0.7
experiment 1
experiment 2
b
MDA-MB-231
MCF7
MCF7 + NET 3 h
MCF7 + NET 6 h
MCF7 + NET 12 h
MCF7 + NET 24 h
MCF7
MCF7 + NET 3 h
MCF7 + NET 6 h
MCF7 + NET 12 h
MCF7 + NET 24 h
kDa
260
160
Fibronectin
6.8 1.0 4.8 2.6 6.1 10 1.0 2.1 0.8 7.5 3.3

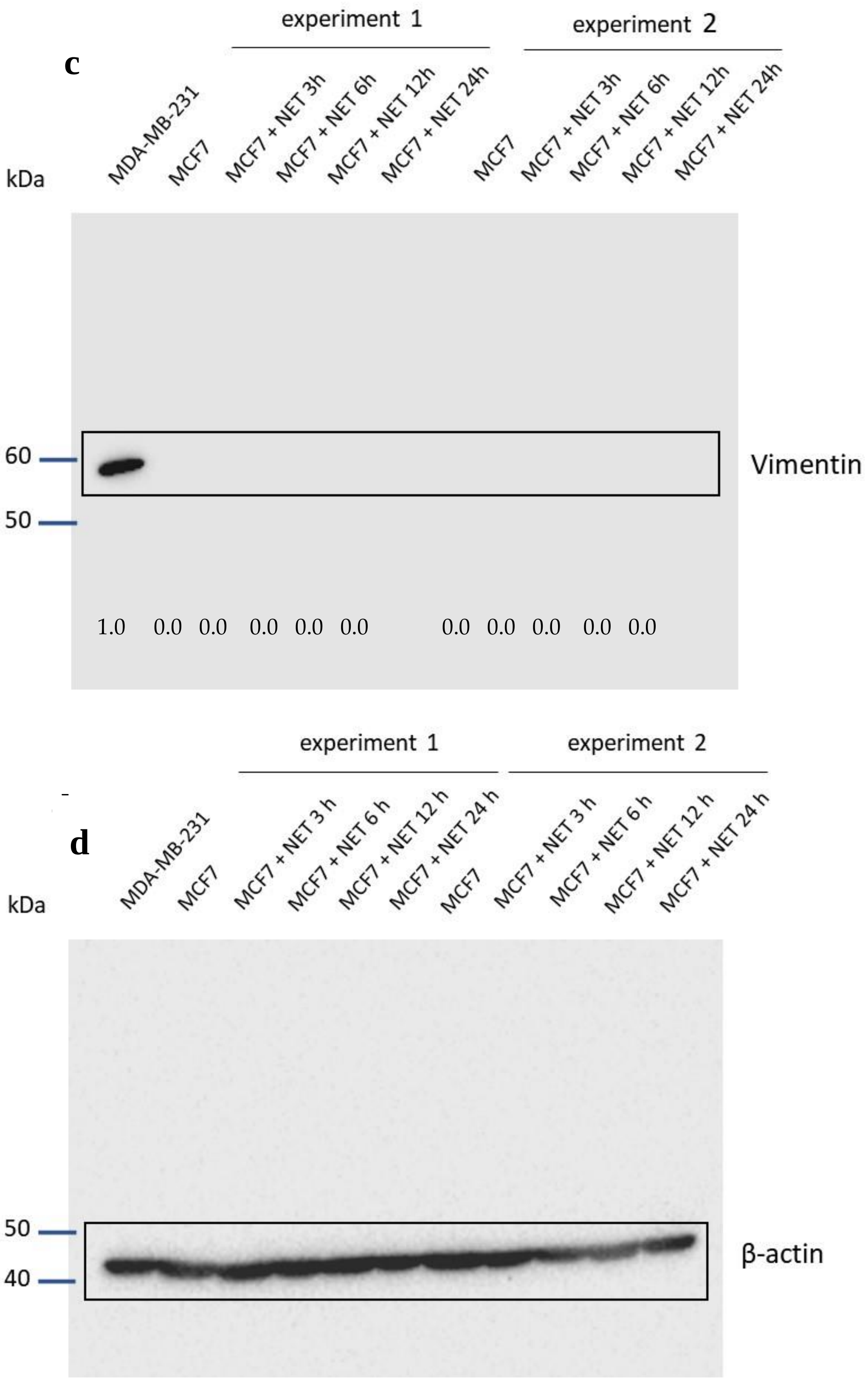
c
experiment 1
experiment 2
kDa
MDA-MB-231
MCF7
MCF7 + NET 3h
MCF7 + NET 6h
MCF7 + NET 12h
MCF7 + NET 24h
MCF7
MCF7 + NET 3h
MCF7 + NET 6h
MCF7 + NET 12h
MCF7 + NET 24h
60
50
Vimentin
1.0 0.0 0.0 0.0 0.0 0.0 0.0 0.0 0.0 0.0 0.0
d
experiment 1
experiment 2
kDa
MDA-MB-231
MCF7
MCF7 + NET 3 h
MCF7 + NET 6 h
MCF7 + NET 12 h
MCF7 + NET 24 h
MCF7
MCF7 + NET 3 h
MCF7 + NET 6 h
MCF7 + NET 12 h
MCF7 + NET 24 h
50
40
β-actin

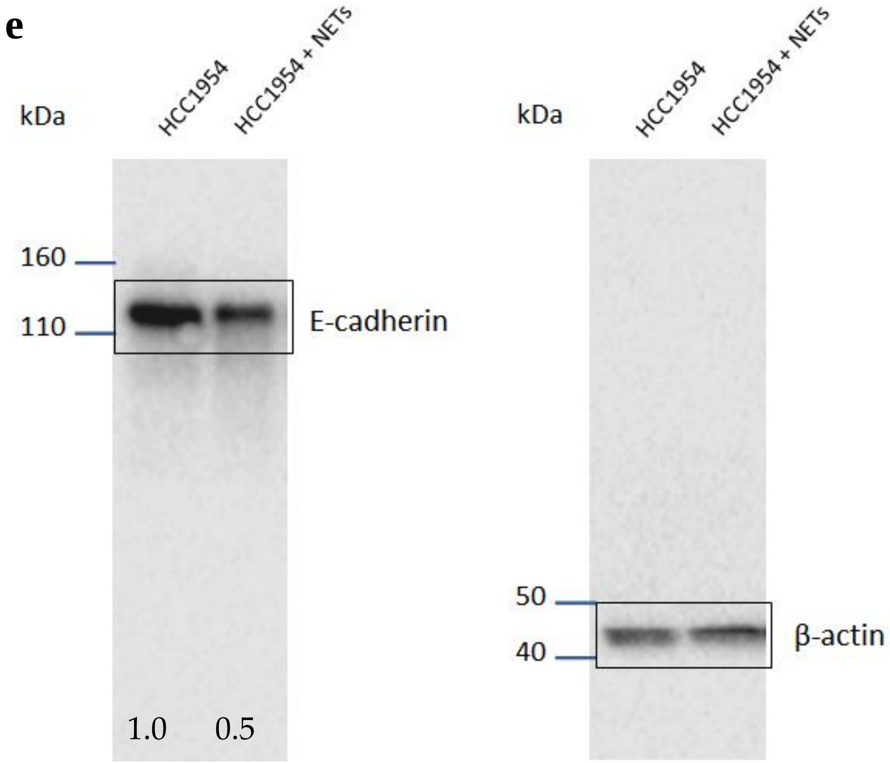

Figure S1. Uncropped blots for analysis of EMT markers. MCF7 cells were starved and treated with NETs for 3 to 24 hours. MDA-MB-231 cells (MDA) were used as a mesenchymal cell model. The figure shows the uncropped blots revealed with antibodies against (**a**) E-cadherin, (**b**) fibronectin, (**c**) vimentin, or (**d**) β-actin, which was used as the loading control. Also, (**e**) E-cadherin levels were analyzed in HCC 1954 cells treated with NETs, using β-actin as a loading control. Densitometric analysis was performed with the ImageJ software (NIH, USA). Fold difference was calculated in relation to untreated MCF7 cell line (**a**,**b**), MDA-MB-231 cells (**c**), or untreated HCC 1954 cells (**e**) depicted at the bottom of each blot. Molecular weight was determined using the Novex™ Sharp Pre-stained Protein Standard (#LC5800, ThermoFisher Scientific). Experiment #1 and experiment #2 refer to two independent assays.

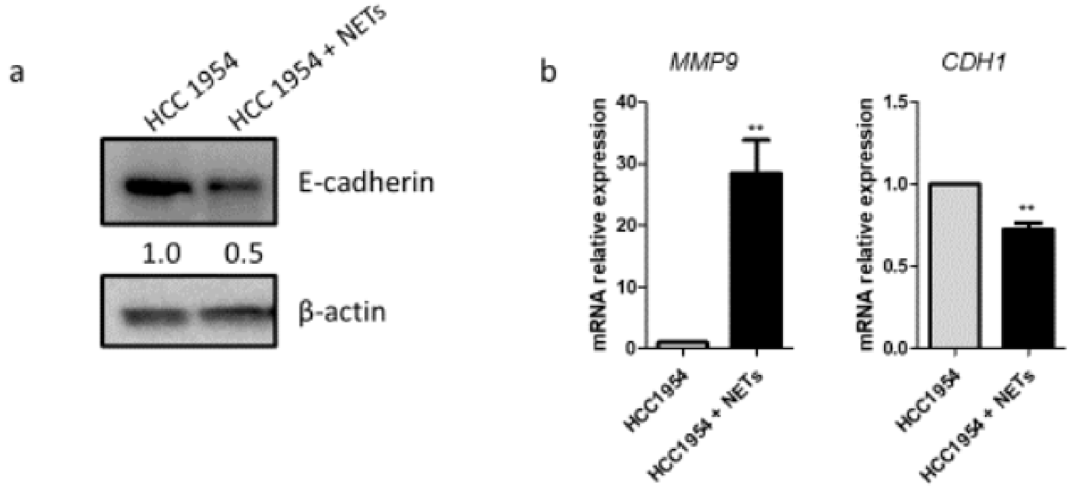

Figure S2. Effect of NETs on HER2+ breast cancer cells. HCC 1954 cells were treated with NETs (500 ng/mL) for 16 h (gene expression analyzes) or 24h (western blot). (**a**) Gene expression of *MMP9* and E-cadherin (*CDH1*) was evaluated by quantitative RT-PCR using the ΔΔCT method. *GAPDH* was used as the reference gene. Columns represent means ± SD of three independent experiments. ** denotes $p < 0.01$ (unpaired *t*-test). (**b**) Protein levels of E-cadherin and β-actin (loading control) were evaluated by western blotting.

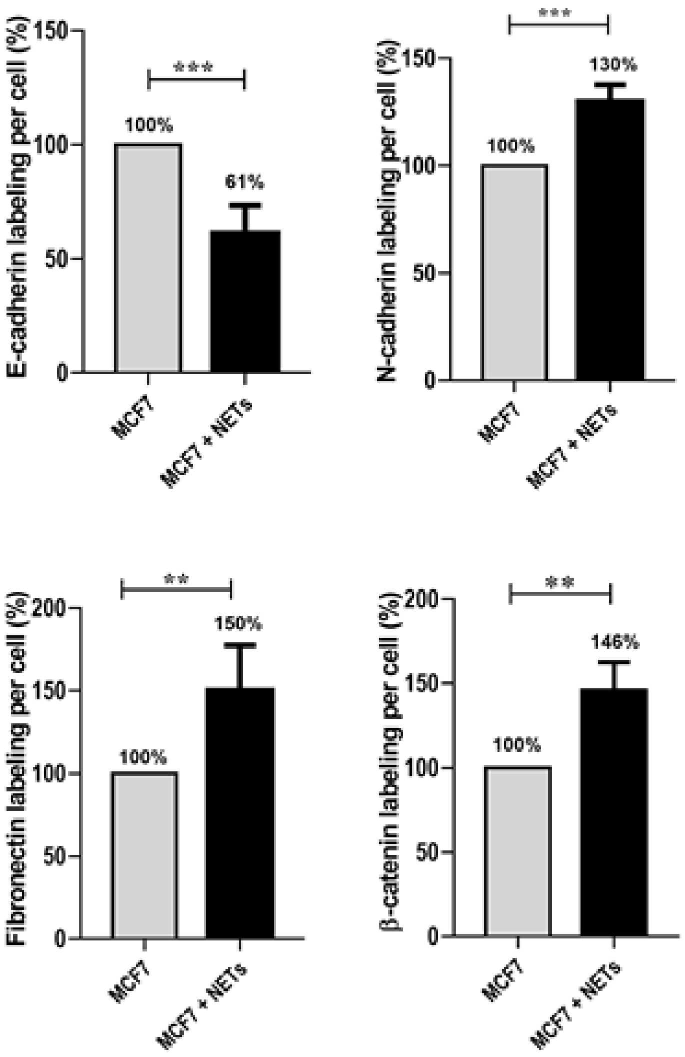

Figure S3. Quantitative analysis of immunocytochemistry assays for EMT markers in NETs-treated MCF7 cells. Quantification of the immunostaining shown in the Figures 2c–f was performed using the ImageJ software. The unpaired *t*-test was applied as a statistical method. ** $p < 0.01$, *** $p < 0.001$.

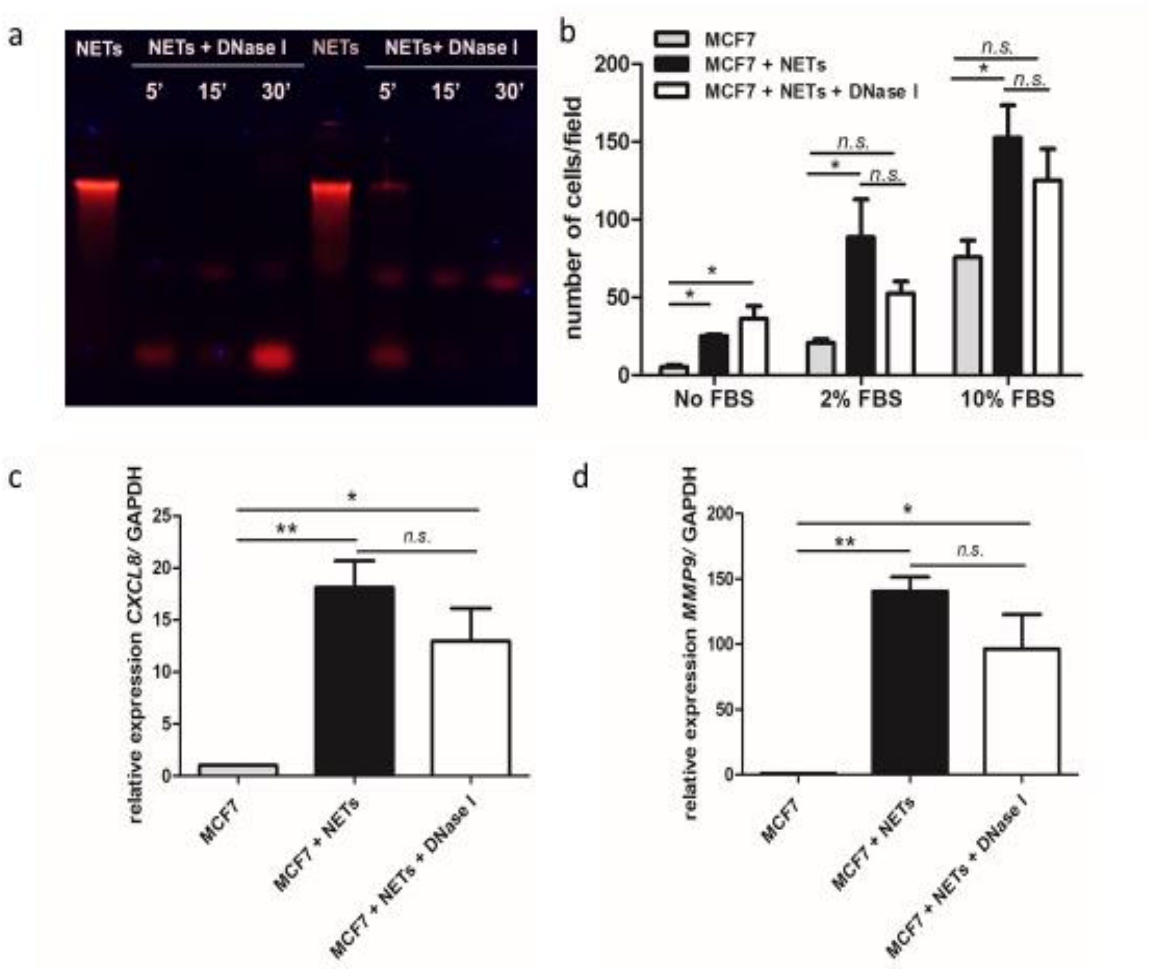

Figure S4. The pro-tumoral effects of NETs are independent of DNA integrity. (**a**) The digestion of NETs was evaluated by agarose gel electrophoresis. NETs, prepared from two distinct healthy donors, were incubated with 5 U DNase I (Pulmozyme®, Roche, Basel, Switzerland), at 37 °C, for the indicated times. (**b**) Tumor cell migration was evaluated employing the Boyden chamber assay. MCF7 cells that were cultured for 16 h in the absence or the presence of either full or digested (5 U DNase I, 30 min, 37 °C) NETs (500 ng/mL) were seeded in the upper chamber (5 × 10^4 cells/well) and further allowed to migrate for 20 h. As chemoattractant, medium supplemented with FBS (2% or 10%) was used in lower chambers. Data are presented as mean ± SD from three independent experiments. Statistical analysis of each condition was evaluated by unpaired *t*-test. * $p < 0.05$, *n.s.*, no significance. (**c**,**d**) MCF7 cells were treated for 16 h with either full or digested NETs (500 ng/mL). Gene expression of IL-8 (*CXCL8)* and *MMP9* was evaluated by quantitative RT-PCR using the ΔΔCT method. *GAPDH* was used as the reference gene. Columns represent means ± SD of three independent experiments. Statistical analysis was performed using one-way ANOVA and Tukey post-test. * $p < 0.05$, ** $p < 0.01$, *n.s.*, no significance.

Table S1. qRT-PCR primer sequences.

Primer	Forward primers (5′–3′)	Reverse primers (5′–3′)	Size bp
GAPDH	5′- TGCACCACCAACTGCTTAGG -3′	5′- GGCATGGACTGTGGTCATGAG -3′	87
CXCR1	5′- CGTCTGTCAATGTCTCTTCCAACC -3′	5′- GATAGTGCCTGTCCAGAGCCAG -3′	127
IL1B	5′- GGACAGGATATGGAGCAACAA -3′	5′- TCTTTCAACACGCAGGACAG -3′	128
IL6	5′- TACCCCAGGAGAAGATTCC - 3′	5′- TTTTCTGCCAGTGCCTCTTT -3′	174
CXCL8	5′- CTGGACCCCAAGGAAAACTG -3′	5′- GAATTCTCAGCCCTCTTCAAAAAC -3′	65
MMP2	5′- AGCTCCCGGAAAGAGTTGATG -3′	5′- CAGGGTGCTGGCTGAGTAGAT -3′	101
MMP9	5′- GCAATGCTGATGGGAAACCC -3′	5′- AGAAGCCGAAGAGCTTGTCC -3′	144
TWIST1	5′- CCGGAGACCTAGATGTCATT -3′	5′- CACGCCCTGTTTCTTTGAA -3′	148
SNAI1	5′- TCG GAA GCC TAA CTA CAG CGA-3′	5′- AGA TGA GCA TTG GCA GCG AG -3′	140
SNAI2	5′- AAG CAT TTC AAC GCC TCC AAA -3′	5′- GGA TCT CTG GTT GTG GTA TGA CA -3′	118
ZEB1	5′- TGGAATGTATGCTTGTGATTTGTG -3′	5′- GAATAAGACCCAGAGTGTGAGAAG -3′	225
ZEB2	5′- CCC TTC TGC GAC ATA AAT ACG A -3′	5′-TGT GAT TCA TGT GCT GCG AGT -3′	192
CD24	5′- CCCACGCAGATTTATTCCAG -3′	5′- GACTTCCAGACGCCATTTG -3′	255
CD44	5′- GGAGCAGCACTTCAGGAGGTTAC -3′	5′- GGAATGTGTCTTGGTCTCTGGTAGC -3′	129
PTGS2	5′- TGGTGCCTGGTCTGATGATG -3′	5′- GCCTGCTTGTCTGGAACAAC -3′	120
